# Non-invasive brain stimulation for fibromyalgia: current trends and future perspectives

**DOI:** 10.3389/fnins.2023.1288765

**Published:** 2023-10-19

**Authors:** Jia-Hao Zhang, Jian Liang, Zhong-Wei Yang

**Affiliations:** ^1^Laboratory of Laser Sports Medicine, School of Physical Education and Sports Science, South China Normal University, Guangzhou, China; ^2^Laboratory of Sports Rehabilitation, School of Physical Education and Sports Science, South China Normal University, Guangzhou, China

**Keywords:** non-invasive brain stimulation (NIBS), fibromyalgia (FM), repetitive transcranial magnetic stimulation (rTMS), transcranial direct current stimulation (tDCS), electroconvulsive therapy (ECT), transcranial alternating current stimulation (tACS), reduced impedance non-invasive cortical electrostimulation (RINCE), transcranial focused ultrasound (tFUS)

## Abstract

Fibromyalgia, a common and enduring pain disorder, ranks as the second most prevalent rheumatic disease after osteoarthritis. Recent years have witnessed successful treatment using non-invasive brain stimulation. Transcranial magnetic stimulation, transcranial direct current stimulation, and electroconvulsion therapy have shown promise in treating chronic pain. This article reviews the literature concerning non-invasive stimulation for fibromyalgia treatment, its mechanisms, and establishes a scientific basis for rehabilitation, and discusses the future directions for research and development prospects of these techniques are discussed.

## 1. Introduction

Fibromyalgia (FM) stands as a common chronic pain disorder (Giorgi et al., 2023), ranking second among rheumatic diseases after osteoarthritis (Clauw, 2014). Its prevalence remains consistent across races, approximately ranging from 2% to 4% (Sarzi-Puttini et al., 2020), with a female-to-male ratio of 7:1 (Neumann and Dan, 2003; Weir et al., 2006). FM is associated with various comorbidities (Robinson et al., 2004) and impacts global quality of life (Clauw, 2014; Chinn et al., 2016), though its pathogenesis remains partially understood (Sarzi-Puttini et al., 2020), implicating central sensitization, neurotransmitter imbalances, neurofunctional irregularities, and endocrine metabolic disturbances.

Pharmacological treatment remains predominant for FM; however, evidence suggests inadequacies in symptom alleviation and adverse reactions in some patients (Mease et al., 2013; Arnold et al., 2015; Gilron et al., 2016). Traditional analgesics, such as acetaminophen, yield inefficacy and severe side effects, leading some to consider antipsychotics for sleep improvement (Moldofsky et al., 1996). While quetiapine shows advantages in pain and sleep issues, its recommendation the level of evidence remains limited, suitable primarily for short-term FM treatment (Calandre et al., 2014; Walitt et al., 2016). A meta-analysis indicates partial symptom relief from drugs like amitriptyline, growth hormone, and sodium oxybate (Perrot and Russell, 2014). In summary, pharmacological treatments inadequately address FM symptoms, necessitating improved approaches (Rathore and Afridi, 2020). With the recognition of central pain system abnormalities in FM’s occurrence, non-invasive brain stimulation (NIBS) techniques emerge as potential non-pharmacological treatments (Fregni et al., 2006; Sampson et al., 2006; O'brien et al., 2018).

Non-invasive brain stimulation (NIBS), widely employed in treating depression, exhibits significant therapeutic effects. The co-occurrence of chronic pain and depression, likely stemming from functional impairment due to persistent pain (Thompson et al., 2016; Michaelides and Zis, 2019), underscores the potential of NIBS as a valuable intervention. Shared neuroanatomical structures and neurochemical phenomena further establish the link between depression and pain (Banks Sara and Kerns Robert, 1996), rendering NIBS not only applicable to depression but also to fibromyalgia (FM).

**Figure 1:**
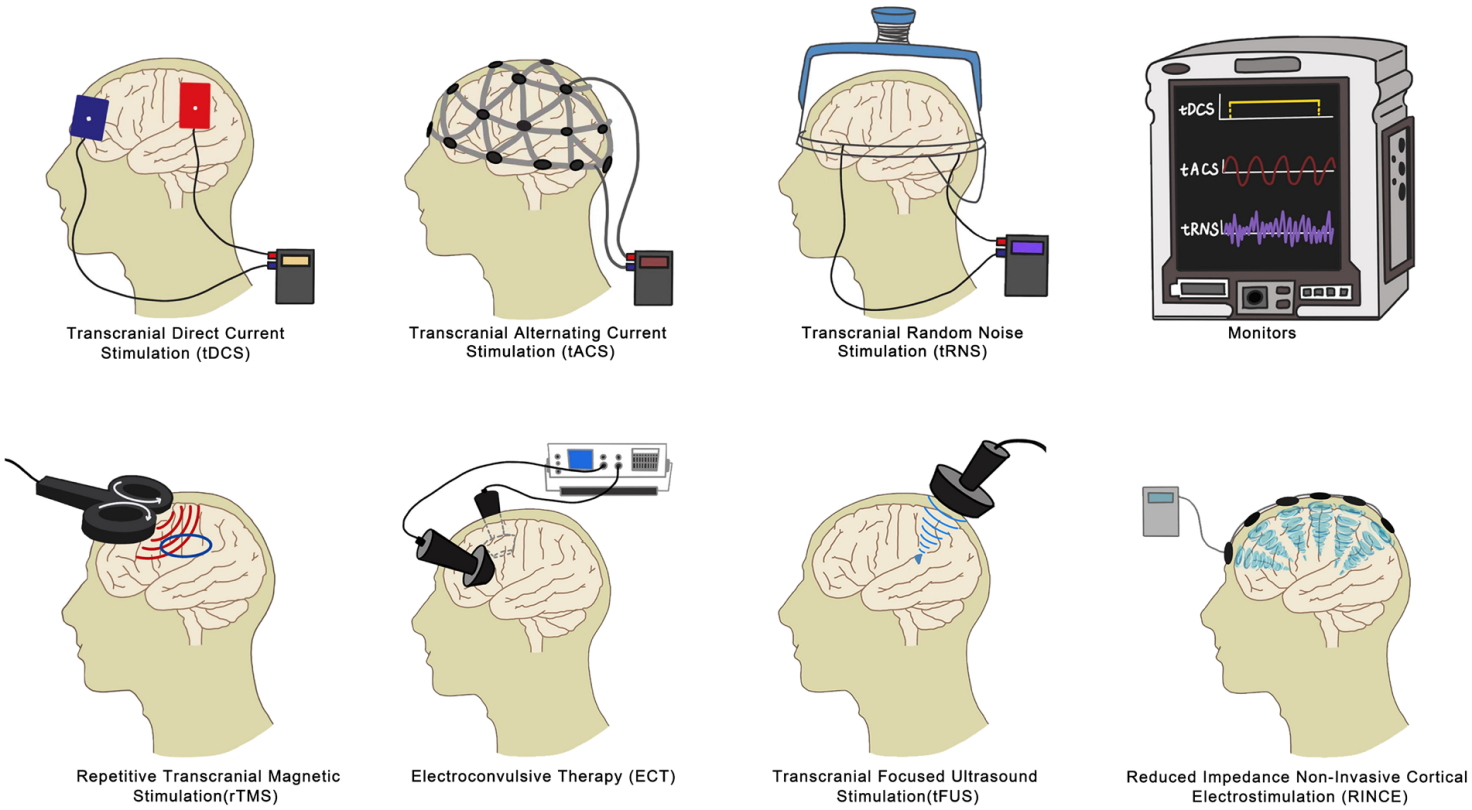
A brief schematic of different non-invasive brain stimulation methods of action.

Among the commonly used and extensively studied NIBS techniques are repetitive transcranial magnetic stimulation (rTMS), transcranial direct current stimulation (tDCS), and electroconvulsive therapy (ECT). These techniques have shown efficacy in addressing FM symptoms, notably improving pain, fatigue, and sleep issues. However, there are other emerging branches of NIBS technology, such as Transcranial Alternating Current Stimulation (tACS), Reduced Impedance Non-Invasive Cortical Electrostimulation (RINCE), Transcranial Focused Ultrasound (tFUS), and Transcranial Random Noise Stimulation (tRNS). These methods possess their own unique characteristics and advantages. While they have fewer related studies compared to transcranial magnetic stimulation, tDCS, and electroconvulsive therapy, they hold significant promise for the future of FM relief and warrant further exploration. In this review, we focus on rTMS, tDCS, and ECT, delving into their mechanisms, clinical effects, and distinctions. This comprehensive examination establishes a solid scientific foundation for the rehabilitation of FM (Figure 1).

## 2. The role of repetitive transcranial magnetic stimulation in fibromyalgia

### 2.1. Introduction to repetitive transcranial magnetic stimulation (rTMS)

Repetitive Transcranial Magnetic Stimulation (rTMS) employs an inductor and a capacitor to generate changing magnetic fields. These fields induce a reverse current in specific brain regions, affecting neuronal functions and electrical activities. The therapeutic impacts of rTMS rely on stimulation parameters, intervention duration, and targeted brain areas. Stimulation intensity typically ranges from 80 to 120% of the participant’s resting motor threshold. rTMS encompasses both excitatory (high-frequency rTMS, HF-rTMS, ≥5 Hz) and inhibitory (low-frequency rTMS, LF-rTMS, ≤1 Hz) modes, provoking neuronal excitation or reduction, respectively (Rosen et al., 2009; Ansari et al., 2021). The multipulse properties of this technique induce physiological neurological changes, which can have lasting effects for up to 15 min or more, even after the stimulation ends (Chen et al., 1997). In the case of fibromyalgia patients, they continued to experience significant pain relief following 10 or more consecutive sessions of transcranial magnetic stimulation, in contrast to the sham stimulation and placebo groups (Boyer et al., 2014; Altas et al., 2019). Notably, analgesic outcomes can extend for weeks (Moisset and Lefaucheur, 2019). In contrast to conventional pharmacological methods, rTMS offers a safer, lower side-effect approach for fibromyalgia treatment. Moreover, rTMS addresses concurrent symptoms in fibromyalgia patients, such as sleep disruptions, fatigue, and functional impairments (Su et al., 2021).

### 2.2. Application and mechanism of rTMS in fibromyalgia treatment

#### 2.2.1. Analgesic effects of rTMS via primary motor cortex and dorsolateral prefrontal cortex

Studies by Tamura et al. (2004) and Sampson et al. (2006) reveal that applying LF-rTMS (1 Hz) to the primary motor cortex (M1) and dorsolateral prefrontal cortex (DLPFC) respectively, alleviates acute pain and offers potential pain regulatory effects in fibromyalgia treatment. Intriguingly, HF-rTMS also contributes to pain relief. Altas’ research demonstrates HF-rTMS (10 Hz) applied to M1 and DLPFC enhances physical function and emotional well-being in fibromyalgia patients (Altas et al., 2019). Furthermore, both high-frequency HF-rTMS and low-frequency LF-rTMS on the right DLPFC manifest analgesic and antidepressant outcomes (Ansari et al., 2021). Collectively, both HF-rTMS and LF-rTMS alleviate fibromyalgia pain through M1 and DLPFC modulation.

#### 2.2.2. Analgesic effects of rTMS via neurotransmitter modulation

rTMS engages cortical neurons, affecting cortical excitability and brain activity to modulate pain processing. Disrupted neurotransmitter concentrations, notably glutamate N-methyl-D-aspartate (NMDA) receptor, contribute to central sensitization and fibromyalgia pain development. rTMS potentially mitigates pain by top-down neurotransmitter modulation (Dall’agnol et al., 2014).

Numerous investigations affirm rTMS’s engagement with the endogenous opioid system, generating analgesic effects. Dysregulation of the endogenous opioid peptide system and diminished opioid receptors characterize fibromyalgia patients (Ansari et al., 2021). Neuroimaging exposes pain-processing regions like the cingulate gyrus, orbitofrontal and prefrontal cortex, thalamus, and periaqueductal gray matter (Peyron et al., 2000; Apkarian et al., 2005). rTMS regulates dopamine, serotonin, and opioid peptide receptors, restoring homeostasis. Further, rTMS on rats’ cerebellar cortex reduces metabolic glutamate receptors and Protein kinase C synthesis, influencing neuronal activity and calcium levels, thus providing analgesic effects (Lee et al., 2014). HF-rTMS might alleviate pain via nitric oxide synthase (nNOS) down-regulation.

FM pain associates with gamma-aminobutyric acid (GABA) and glutamate (Glu) mechanism dysregulation (Mhalla et al., 2010). rTMS of the ipsilateral motor cortex significantly elevates inhibitory neurotransmitter GABA, with the contralateral side experiencing a decrease (Clauw, 2014). Consequently, impaired GABAergic neurotransmission correlates with FM pain. Motor cortex-targeted rTMS reinstates GABAergic and glutamatergic system balance, curbing excitatory and bolstering inhibitory neurotransmitter activity for fibromyalgia pain relief. To summarize, rTMS stands as a secure, non-invasive cortical stimulation technique.

## 3. The role and mechanism of transcranial direct current stimulation treatment in fibromyalgia

### 3.1. Introduction to transcranial direct current stimulation (tDCS)

tDCS employs small, constant currents (1–2 mA) externally to modulate brain neuronal activity. It has gained diverse applications in brain injury rehabilitation, cognitive and emotional regulation, and chronic pain management. Renowned for its affordability and tolerance, tDCS stands as a widely used non-invasive brain stimulation method (Saavedra et al., 2015). The stimulation modes categorize as anodal (a-tDCS), cathodal (c-tDCS), or sham (s-tDCS), each differing in action (Liu et al., 2021). Notably, while transcranial electrical stimulation adjusts neural network activity, transcranial magnetic stimulation triggers neuronal firing via suprathreshold stimulation. At the neuronal level, tDCS achieves effects by polarizing the resting membrane potential through different polarities, thus modulating cortical excitability. Notably, membrane polarization is the primary mechanism for transcranial electrical stimulation, with a-tDCS heightening cortical excitability and c-tDCS inhibiting it. The evidence-based recommendation for the application of tDCS in fibromyalgia is categorized as level B, according to the Standards of the European Federation of Neurological Societies (Lefaucheur et al., 2017).

### 3.2. Application and mechanism of tDCS in fibromyalgia treatment

#### 3.2.1. tDCS and analgesic effects via targeting brain regions

Distinct tDCS types can be employed to mitigate fibromyalgia (FM) pain by focusing on diverse brain regions. Application of c-tDCS on S1, M1, or DLPFC reduces brain hyperexcitability, increasing pain thresholds and reducing sensitivity. However, targeting DLPFC for pain relief in FM patients is somewhat debated (Fregni et al., 2006). Nevertheless, research suggests c-tDCS applied to DLPFC could alleviate pain (Mariano et al., 2015). Valle’s study indicates a-tDCS on left M1 is more effective than left DLPFC in producing analgesic effects (Valle et al., 2009). Additionally, Fregni et al. (2006) find that a-tDCS targeting M1 substantially improves FM pain, sustaining analgesic effects for over 3 weeks. This suggests that M1 targeting is more effective. Still, the analgesic effects of c-tDCS on M1 remain debated (Steffen et al., 2018). Conversely, Roizenblatt et al. (2007) propose tDCS on M1 improves sleep in FM patients. Other studies suggest M1 stimulation could influence thalamus and basal ganglia function, thereby alleviating FM pain (Khedr et al., 2017). In summary, targeting M1 with tDCS seems more effective in alleviating FM pain symptoms.

#### 3.2.2. tDCS and analgesic effects via neurotransmitter modulation

The analgesic effects of tDCS relate to neurotransmitter alterations. Studies underscore NMDA receptor activation’s role in pain generation and maintenance, a potential mechanism in fibromyalgia (FM). NMDA receptor non-competitive antagonist MK-801 reduces capsaicin-induced heat hyperalgesia and mechanical hypersensitivity. During tDCS, changes in membrane potential influence NMDA receptor expression and enhance γ-aminobutyric acid (GABA) release, thus easing pain (Foerster et al., 2015). N-acetylaspartate (NAA), an abundant brain metabolite, serves as a neuroprotective neurotransmitter, significant in pain management. Bradley R. Foerster et al. find tDCS decreases clinical pain scores, reduces glutamate and glutamine levels, and increases γ-GABA, hinting at enhanced neurotransmitter modulation to ease pain. Furthermore, tDCS elevates N-acetylaspartate levels, overall impacting neurotransmitters for pain relief. In addition, Kadetoff et al. (2012) observed elevated interleukin-8 (IL-8) in the cerebrospinal fluid of fibromyalgia (FM) patients, which is likely pivotal in the central sensitization process.

## 4. The role of electroconvulsive therapy in fibromyalgia treatment

### 4.1. Introduction to electroconvulsive therapy (ECT)

ECT involves applying electrical potential to the brain via stimulation electrodes affixed to the scalp, creating diverse electric fields based on electrode positioning. This induces synchronized neural cell oscillations and simultaneous autonomic nervous system excitation across extensive brain areas (Couvreur et al., 1989; Peterchev et al., 2010). Since Cerletti and Bini’s initial use of ECT for schizophrenia treatment in 1938(Kalinowsky, 1986), physicians have applied it to various conditions. Rasmussen et al. reviewed ECT’s pain treatment literature, concluding its efficacy in chronic pain management based on synthesized case reports spanning decades (Rasmussen and Rummans, 2002). Moreover, reports indicate ECT’s analgesic effects across different pain syndromes (Canavero and Bonicalzi, 2001; Wasan et al., 2004).

### 4.2. Application and mechanism of ECT in fibromyalgia treatment

#### 4.2.1. ECT and analgesic effects via enhanced cerebral blood flow

Usui et al.’s study tracked regional cerebral blood flow (rCBF) changes pre and post ECT, revealing thalamic rCBF increase in fibromyalgia (FM) patients. This correlated with reduced tender points and pain scores (visual analog scale, VAS), indicating significant pain relief possibly tied to augmented rCBF (Usui et al., 2006). Neuroimaging verifies the prefrontal cortex, anterior cingulate cortex, insula, and amygdala’s roles in pain and emotion regulation (Rainville, 2002). Severe depression and chronic pain patients exhibit abnormal blood flow in these regions (Marsh et al., 1996), which ECT can normalize in severe depression (Elizagarate et al., 2001) and chronic pain (Fukui et al., 2002b). While some studies suggest ECT’s efficacy in low thalamic blood flow neuropathic pain (Fukui et al., 2002a, 2002b), other studies report mixed results (Salmon et al., 1988, McCance et al., 1996). Notably, ECT could raise brain-derived neurotrophic factor (BDNF) (Okamoto et al., 2008; Piccinni et al., 2009), pivotal in antidepressant actions through neural plasticity and linked to pain improvement.

#### 4.2.2. ECT and analgesic effects via neurotransmitter modulation

ECT heightens responsiveness of serotonin, norepinephrine, and dopamine systems pivotal in central pain processing (King and Liston, 1990). ECT can stimulate inhibitory pathways by activating these neurotransmitter systems (Newman et al., 1998). Post-ECT treatment, neurotransmitter release increases, bolstering serotonin, norepinephrine, dopamine, and others. This enhances peripheral stimulus signal processing through central nervous system descending pathways, reinforcing brain inhibitory pain pathways (Canavero et al., 1994; Wasan et al., 2004).

ECT fosters central neurotransmitter systems’ new equilibrium through electrical stimulation. Past studies indicate repeated ECT boosts plasma beta-endorphin levels (Abrams, 2002). Insufficient beta-endorphin levels correlate with pain hypersensitivity. ECT might relieve fibromyalgia pain by promoting endorphin production, reducing pain sensitivity, and achieving analgesic effects. Okabe et al. note ECT’s potential to increase neuropeptide Y (NPY) expression (Okabe et al., 2010). NPY, a hormone with central and peripheral presence, plays diverse roles, including pain modulation. The mechanism by which ECT treats pain in patients with FM may involve promoting elevated NPY expression, thereby reducing pain sensitivity in patients and achieving analgesic effects.

## 5. Profiles of several non-invasive brain neurostimulation techniques

### 5.1. Transcranial alternating current stimulation (tACS)

tACS, a non-invasive brain stimulation technique, modulates neural activity by applying sinusoidal alternating current to the scalp, thus generating an electric field within the brain (Wischnewski et al., 2023). Much like direct current stimulation (tDCS), tACS effectively modulates cortical excitability (Sabel et al., 2020). However, when compared to tDCS, tACS demonstrates superior efficacy in precisely directing endogenous brain oscillations. It has the unique capability to mimic the natural alternation of brain oscillations and induce long-term synaptic plasticity, thereby effectively regulating brain function.

Despite being a more widely adopted non-invasive brain stimulation method, tACS is still relatively nascent in its development, particularly in the context of fibromyalgia. In recent years, researchers have successfully addressed the traditional limitations of tACS, which struggled with the precise localization of specific brain regions. They have accomplished this through innovative techniques, including high-definition tACS, phase-shifting tACS, amplitude-modulated tACS, temporal interference (TI) techniques, and intersecting short pulses (ISPs) (Wu et al., 2021). These advancements have propelled tACS into a more advanced stage of development and have expanded its potential in the treatment and alleviation of fibromyalgia symptoms.

### 5.2. Reduced impedance non-invasive cortical electrostimulation (RINCE)

RINCE is among the less explored methods of electrical stimulation within the realm of non-invasive brain stimulation. In RINCE, electrodes attached to the patient’s scalp generate a specific current frequency, allowing the current to penetrate deeper into the cortex by reducing the impedance of the skin and skull (O'Connell et al., 2018), potentially leading to improved stimulation effects. There are limited reports on RINCE therapy, and its adverse effects include transient mild head discomfort and localized cutaneous reactions (Szymoniuk et al., 2023). In an RCT investigating RINCE therapy for fibromyalgia control, it was found that mean pressure points and pressure pain thresholds improved in the active treatment group compared to the sham treatment group (Hargrove et al., 2012). Patients with fibromyalgia reported decreased pain VAS scores following RINCE treatment. Although there is limited current research on RINCE, these results are statistically and clinically significant, suggesting that RINCE offers advantages and potential for development in fibromyalgia treatment.

### 5.3. Transcranial focused ultrasound (tFUS)

tFUS emerges as a significant non-invasive brain stimulation technique (Aubry and Tanter, 2016). While it may not have received the same level of research attention as rTMS, tDCS, or other established methods, it boasts remarkable spatial precision, capable of targeting and stimulating deep brain regions with millimeter accuracy. This technique employs piezoelectric-element transducers that emit ultrasound pulses, effectively reaching and stimulating deep brain areas.

### 5.4. Transcranial random noise stimulation (tRNS)

tRNS, a non-invasive brain stimulation method, is renowned for its reduced discomfort levels in comparison to other tES techniques. In an analgesic study, tRNS demonstrated the capability to induce both immediate and sustained analgesia when applied to area m1 of the cerebral cortex. This analgesic effect was attributed to a reduction in pain anticipation (Yao et al., 2021). Furthermore, it exhibits potential for enhancing behaviors through long-term neuroplasticity effects (van der Groen et al., 2022). Nonetheless, the precise physiological mechanisms responsible for tRNS’s impact on nerves remain elusive.

## 6. Discussion

Limited studies have investigated the effects of tRNS, RINCE, and tFUS on fibromyalgia compared to tACS. In a double-blind randomized crossover study conducted by Bernardi et al., participants were randomly assigned to receive either transcranial alternating current stimulation (tACS) or random noise stimulation (RNS) treatment 5 days a week for 2 weeks. The intervention group received tACS, while the control group received RNS. The study defined three measurement time points: T0 (baseline), T1 (post-stimulation), and T2 (1 month or 4 weeks after stimulation) (Bernardi et al., 2021).

The results showed that, in comparison to RNS, tACS led to an increase in EEG alpha1 activity [(8–10) Hz] at T1, a reduction in pain symptoms assessed through visual analog scales at T1, and enhancements in self-reported cognitive skills and neuropsychological scores at both T1 and T2. Notably, improvements were observed in both the tACS and RNS groups after receiving treatment. However, it’s important to note that this study had a small sample size and potential bias due to variations in participants’ medication regimens. To address this limitation, future studies should aim to validate this treatment approach using larger samples. Additionally, maintaining consistent medication regimens among study participants throughout the research is essential to mitigate potential bias from concurrent medication use.

## 7. Conclusion and future perspectives

The emergence of non-invasive brain stimulation techniques has revolutionized chronic pain treatment. Methods like rTMS, tDCS, and ECT have proven effective in addressing conditions such as fibromyalgia, showcasing considerable potential. Lesser-known techniques like rINCE, tFUS, tACS, and RNS, while having fewer studies behind them, exhibit promising traits and are poised for development. This paper delves into the intricacies of non-invasive brain stimulation mechanisms, identifies technical challenges, and outlines future research directions.

However, research on the application of non-invasive brain stimulation for fibromyalgia and analogous chronic pain conditions is still in its infancy. Several crucial considerations should guide our future endeavors:

1. The current clinical sample size for non-invasive brain stimulation in fibromyalgia remains relatively small. It is imperative to conduct comprehensive, large-scale, and standardized randomized controlled trials to determine optimal stimulation parameters and models for various non-invasive brain stimulation techniques (Hou et al., 2016).
2. Enhancing the credibility of experimental data necessitates supplementing subjective rating scales with objective measures. Furthermore, maintaining the integrity of participant blinding is of paramount importance.
3. An in-depth exploration is vital to pinpoint precise stimulation sites and understand how distinct parameters and target locations influence treatment outcomes. Utilizing neuroimaging and high-resolution electroencephalography can enhance localization, while integrating neurophysiological markers and diverse imaging modalities can refine the determination of stimulation intensity and duration.
4. Recognizing the synergy between non-invasive brain stimulation and pharmacology in fibromyalgia treatment is essential, given the frequent combination of these approaches. This synergy holds the promise of heightened therapeutic efficacy and adherence to rigorous technical standards, optimizing pain relief for fibromyalgia patients.
5. Currently, RINCE, tACS, tFUS, and tRNS, as branches of NIBS technology, have a relatively small number of studies compared with rTMS, tDCS, and ECT, and subsequent researchers have paid more attention to their development and deeper mechanistic studies. These developments are not only conducive to the overall development of non-invasive brain stimulation but will also play a greater potential role in the treatment of fibromyalgia disorders.
6. In light of the unique analgesic mechanisms of various non-invasive stimulation techniques, future research should explore their integration. This approach can overcome individual limitations, paving the way for personalized non-invasive stimulation methods and precision medicine. Such advancements will significantly contribute to the treatment of fibromyalgia and chronic pain, ultimately enhancing patient well-being and quality of life.

## Author contributions

J-HZ: Writing – original draft, Writing – review & editing, Conceptualization, Data curation, Formal Analysis, Validation. JL: Conceptualization, Supervision, Validation, Writing – review & editing. Z-WY: Conceptualization, Supervision, Validation, Writing – review & editing.
